# LncRNA DANCR functions as a competing endogenous RNA to regulate RAB1A expression by sponging miR-634 in glioma

**DOI:** 10.1042/BSR20171664

**Published:** 2018-02-02

**Authors:** Dawei Xu, Jian Yu, Guojun Gao, Guangjian Lu, Yi Zhang, Pengju Ma

**Affiliations:** 1 Department of Neurosurgery, The First Affiliated Hospital of Xinxiang Medical University, Weihui, Henan 453100, China; 2 Department of Pathology, The First Affiliated Hospital of Xinxiang Medical University, Xinxiang, Henan 453000, China; 3 Clinical Laboratory, The First Affiliated Hospital of Xinxiang Medical University, Weihui, Henan 453100, China; 4 Department of Neurology, The First Affiliated Hospital of Xinxiang Medical University, Weihui, Henan 453100, China

**Keywords:** DANCR, Glioma, lncRNA, miR-634, RAB1A

## Abstract

Long noncoding RNA (lncRNA) differentiation antagonizing nonprotein coding RNA (DANCR) plays important regulatory roles in many solid tumors. However, the effect of DANCR in glioma progression and underlying molecular mechanisms were not entirely explored. In the present study, we determined the expression of DANCR in glioma tissues and cell lines using qRT-PCR and further defined the biological functions. Furthermore, we used luciferase reporter assay, Western blot, and RNA immunoprecipitation (RIP) to explore the underlying mechanism. Our results showed that DANCR was significantly up-regulated in glioma tissues and cell lines (U251, U118, LN229, and U87MG). High DANCR expression was correlated with advanced tumor grade. Inhibition of DANCR suppressed the glioma cells proliferation and induced cells arrested in the G0/G1 phase. In addition, we verified that DANCR could directly interact with miR-634 in glioma cells and this interaction resulted in the inhibition of downstream of RAB1A expression. The present study demonstrated that DANCR/miR-634/RAB1A axis plays crucial roles in the progression of glioma, and DANCR might potentially serve as a therapeutic target for the treatment of glioma patients.

## Introduction

Glioma is the most lethal and common type of primary malignant brain tumor in the central nervous system of adults with an annual incidence of five cases per 100,000 people [1]. Glioma is further classified as low-grade glioma (WHO grade I and II) and high-grade glioma (WHO grade III and IV) based on the degree of malignancy according to their histopathologic characteristics [2]. Although combined with surgical resection and postoperative radiochemotherapy, the median survival time for patients with glioma remains distal [3]. Consequently, a better understanding of the molecular mechanisms associated with glioma formation and development is necessary to highlight novel therapeutic targets and develop strategies for the treatment of glioma.

Long noncoding RNA (lncRNA) is nonprotein coding transcripts more than 200 nucleotides (nt) in length [4]. LncRNA has been confirmed to act as key molecules in the progression of various human tumors [5,6]. Differentiation antagonizing nonprotein coding RNA (DANCR) is located on human chromosome 4, with the closest adjacent annotated genes USP46 and ERVMER34-1 [7]. Recent studies indicated that DANCR was up-regulated in a large variety of cancers and thereby of high diagnostic value for great clinical value for cancer therapy. However, the expression and the underlying roles of DANCR in glioma are still unclear.

MicroRNAs (miRNAs) are tiny, noncoding, endogenous, single-stranded RNAs (18–25 nt) that regulate gene expression [8]. Increasing evidence indicated that miRNAs were involved in initiation and progression of cancers as well as glioma [9,10]. miR-634 is a big, poorly conserved primate-specific miRNA family. Recent studies demonstrated that miR-634 play important roles in tumor progression. For example, Cong et al. [11] showed that miR-634 decreased cell proliferation and induced apoptosis by targeting mTOR signaling pathway in cervical cancer cells. Peng et al. [12] showed that miR-634 sensitized nasopharyngeal carcinoma cells to paclitaxel and inhibited cell growth both *in vitro* and *in vivo*. Zhang et al. [13] found that miR-634 exhibited antitumor activities toward hepatocellular carcinoma via RAB1A and DHX33. However, the role of miR-634 in glioma remains unknown.

Recent studies demonstrated that lncRNAs function as competing endogenous RNAs (ceRNA) by sponging miRNAs to regulate gene expression at a post-transcriptional level [14]. Thus, in the present study, we hypothesized that lncRNA DANCR contributed to glioma progression by function as a miRNA sponge. Our study discovered a novel mechanism that DANCR served as a molecular sponge for miR-634 and regulated RAB1A in glioma progression.

## Materials and methods

### Clinical specimens

About 47 glioma samples were obtained from patients who experienced surgical resection from January, 2015 to October, 2016 at the Department of Neurosurgery, The First Affiliated Hospital of Xinxiang Medical University. Normal brain (NB) tissues were obtained from 14 individuals who died in traffic accidents from 2014 to 2016 at the Department of Neurosurgery. None of the patients had received radiotherapy or chemotherapy before surgery. This project was approved by the Ethic Committee of Xinxiang Medical University. All tissues were immediately stored in liquid nitrogen until the total RNA was extracted.

### Cell culture and transfection

Human glioma cell lines, U251, U118, LN229, and U87MG, as well as normal human astrocytes (NHA) were purchased from the Chinese Academy of Sciences (Shanghai, China). The cells were cultured in RPMI-1640 medium supplemented with 10% fetal bovine serum (FBS) and 100 mg/ml at 37°C under a humidified atmosphere of 5% CO_2_.

All the constructs used to knockdown DANCR were purchased from RiboBio (RiboBio). The siRNA sequences were as follows: si-DANCR-1: CTACAGGCACAAGCCATTG; si-DANCR-2: GCGTACTAACTTGTAGCAA [7]. miRNA mimics and miRNA inhibitors were synthesized by Genepharma Company (Shanghai, China). Cell trasfection was conducted by using the Lipofectamine™ 2000 transfection reagent (Invitrogen) according to the manufacturer’s instructions.

### RNA isolation and qRT-PCR

Total RNA was isolated using the Trizol reagent (Invitrogen), followed by removal of DNA with the TurboDNase Kit (Ambion). Quantification of extracted RNA was performed using NanoDrop. cDNA synthesis was performed using PrimeScriptRT reagent KIT (Takara) using 1000 ng of total RNA. QRT-PCR was performed using the SYBR Select Master Mix (Applied Biosystems) on an ABI 7900 system (Applied Biosystem). The level of GAPDH was used as a control. The *C*_t_ value was calculated based on the ΔΔ*C*_t_-method. Fold change of gene expression was expressed as 2^−ΔΔ*C*^_t_ method. The sequences of the PCR primers are as following: DANCR, forward: 5′-GCCACTATGTAGAGGGTTTC-3′ and reverse: 5′-ACCTGCGCTAAGAA-CTGAGG-3′; RAB1A, forward: 5′-GGGAAAACAATCAAGCTTCAAA-3′ and reverse: 5′-CTGGAGGTGATTGTTCGAAAT-3′; GAPDH, forward: 5′-AGAAGGCTGGGGCTCATTTG-3′ and reverse: 5′-AGGGGCCATCCACAGTCTTC-3′.

### Cell proliferation assay

Cell Counting Kit-8 (Dojindo) assay was used to detect the proliferation ability of the cells. The cells were seeded in 96-well plates at 5 × 10^3^ cells/well and were treated under different transfection conditions. At 24, 48, 72, and 96 h post-transcription, 20 μl of CCK-8 was added to each well. The absorbance at 570 nm of each sample was measured by Elx800 (BioTek). Cell viability was calculated according to the absorbance values.

### Colony formation assay

The transfected cells were seeded in six-well plates with culture medium containing 10% FBS and cultured overnight. After 14 days, cells were fixed with methanol and stained with 0.1% Crystal Violet. Colonies were manually counted under a light microscope.

### Flow cytometry

Flow cytometry was performed for cell cycle analysis. Transfected cells were seeded in six-well plates and washed with phosphate-buffered saline (PBS) twice. Cells were fixed with 75% ethanol overnight at 4°C. Cells were then resuspended in 200 μl of PBS with 10 μl of propidium iodide (PI) and incubated at room temperature in the dark for 15 min. Cell cycle was analyzed by a FACScan flow cytometer (Becton Dinkinson). The cell phase was analyzed by ModFit LT software.

### Western blotting

Total proteins derived from glioma cells were extracted and the concentration of the protein was detected by BCA protein assay kit (Sigma). Proteins were fractionated by using 10% sodium dodecyl sulfate/polyacrylamide gel electrophoresis (SDS/PAGE; Sigma). The separated protein was transferred to PVDF membrane (Bio-Rad). The membranes were incubated with RAB1A antibody (Cat No. 28566; Santa Cruz Biotechnology), and GAPDH (Cat No. 25778; Santa Cruz Biotechnology) at 4°C overnight. Next, the blotted membranes were incubated with HRP-conjugated secondary antibody at room temperature for 2 h. Signals were visualized using ECL Substrates (Millipore).

### Dual-luciferase reporter assay

The sequence of DANCR was amplified by PCR, and a mutated (Mut) sequence of DANCR with mutations at the binding site of miR-634 was produced by GeneArt™ Site-Directed Mutagenesis PLUS System (Thermo Fisher Scientific). They were inserted into the pGL3 control vector (Promega). For luciferase assay, the miR-634 mimics or miR-NC were cotransfected with the Wt-DANCR or Mut-DANCR reporter gene plasmid into the HEK293 cells. After transfection for 48 h, dual-luciferase reporter assay system (Promega) was used to determine the luciferase intensity.

### RNA immunoprecipitation (RIP)

The procedure using RIP was carried out using a Magna RNA-binding protein immunoprecipitation kit (Millipore). In brief, whole-cell lysate was incubated with a RIP buffer containing magnetic beads conjugated with human anti-Ago2 antibody. Samples were incubated with Proteinase K and then immunoprecipitated RNA was isolated. In addition, purified RNAs were extracted and analyzed by qRT-PCR to demonstrate the presence of the binding targets.

### Statistical analysis

Statistical analysis was performed using SPSS 16.0 software. All experiments were repeated three times and presented as Mean ± SD. Differences between two groups were estimated using two-tail Student’s *t*-test or one-way ANOVA. Correlations were analyzed by Spearman rank correlation. The differences were considered to be statistically significantly when *P* value is less than 0.05.

## Results

### DANCR expression is up-regulated in glioma

**Figure 1:**
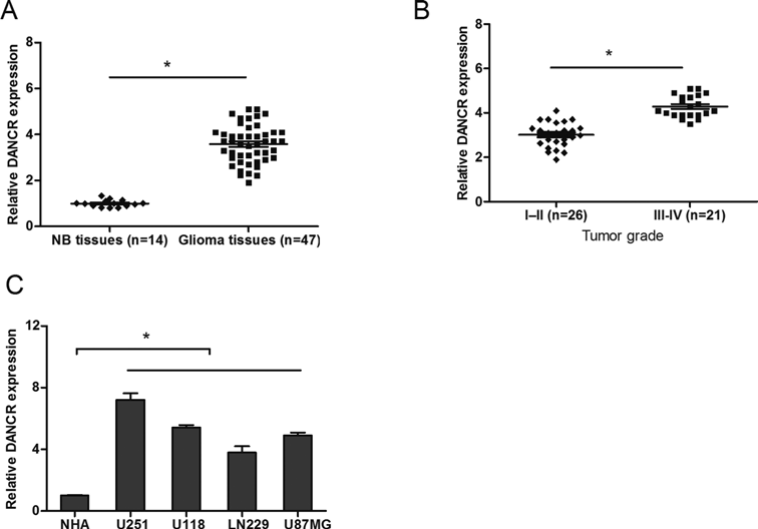
LncRNA DANCR is up-regulated in human glioma tissues and cell lines (**A**) The expression of DANCR in glioma tissues and normal brain (NB) tissues was determined by qRT-PCR. (**B**) DANCR expression in glioma tissues is positively correlated with tumor grades. (**C**) The expression of DANCR in glioma cells (U251, U118, LN229, and U87MG) and normal human astrocytes (NHA) cells was determined by qRT-PCR; *P<0.05.

To identify the role of DANCR in glioma progression, we analyzed the expression of DANCR in 47 glioma tissue samples and 14 normal brain (NB) tissue samples using qRT-PCR. Our data showed that DANCE expression levels were increased in glioma tissue samples compared with NB tissue samples (Figure 1A; *P*<0.05). Furthermore, we found that high DANCR expression levels were positively associated with advanced tumor grade (III-IV) of glioma (Figure 1B; *P*<0.05). We also evaluated the expression of DANCR in glioma cell lines. Results showed that DANCR expression was significantly up-regulated in glioma cell lines (U251, U118, LN229, and U87MG) compared with that in normal human astrocytes (NHA) cells (Figure 1C; *P*<0.05). These data indicated that DANCR overexpression was associated with glioma progression.

### Knockdown of DANCR inhibits glioma cells proliferation

**Figure 2:**
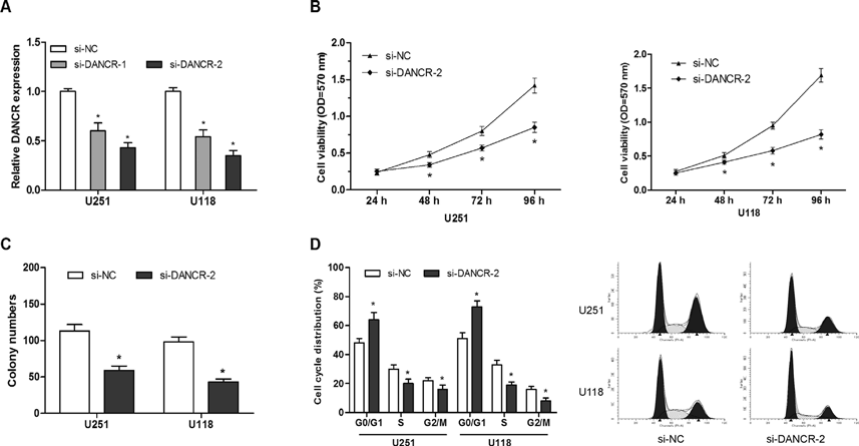
LncRNA DANCR suppression inhibits glioma cells growth *in vitro* (**A**) DANCR mRNA expression in glioma cells after siRNA transfection was determined by qRT-PCR. (**B**) After transfection with the indicated vectors, glioma cells proliferation was determined by CKK-8 assay. (**C**) After transfection with the indicated vectors, colony numbers of glioma cells were determined by colony formation assay. (**D**) After transfection with the indicated vectors, glioma cells cycle was determined by flow cytometry assay; *P<0.05.

To investigate the role of DANCR in glioma progression, we suppressed DANCR expression in glioma cells by transfected siRNAs, and the knockdown efficiency was confirmed by qRT-PCR (Figure 2A; *P*<0.05). CCK-8 assays showed that DANCR inhibition decreased cell proliferation compared with the control group in U251 and U118 cell lines (Figure 2B; *P*<0.05). Colony formation assay showed colony numbers were reduced after cells transfected with si-DANCR than cells transfected with si-NC (Figure 2C; *P*<0.05). To further investigate the role of DANCR on glioma cells cycle, we used flow cytometry. Results showed that DANCR suppression led to a decrease in the percentage of S-phase cells, an increase in the percentage of G0/G1-phase cells (Figure 2D; *P*<0.05). The above findings indicated that DANCR inhibition could suppress glioma cells proliferation by arresting cell cycle in G0/G1 phase.

### DANCR directly targets miR-634 in glioma cells

**Figure 3:**
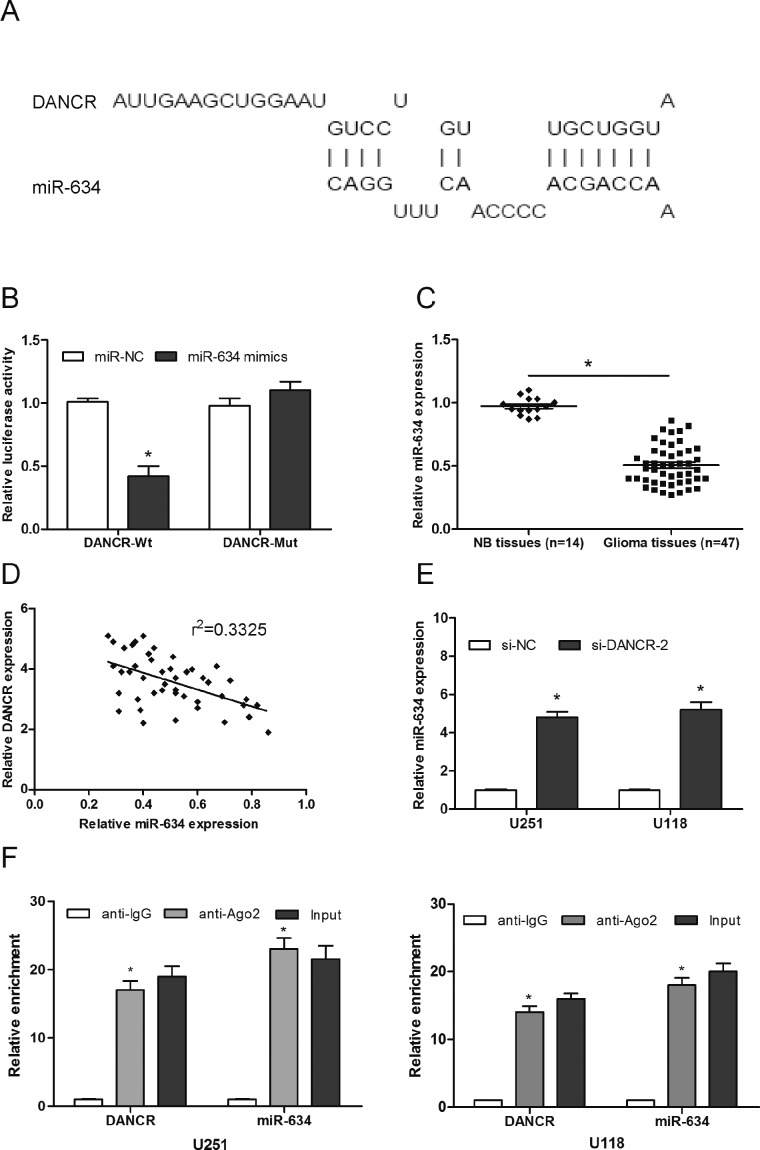
LncRNA DANCR targets miR-634 (**A**) Representation of the miR-634 binding site in DANCR based on DIANA tools. (**B**) Luciferase reporter assay for the targeting of miR-634 to DANCR. (**C**) The expression of miR-634 in glioma tissues and NB tissues was determined by qRT-PCR. (**D**) Negative correlation between DANCR expression and miR-634 expression in 47 glioma tissue samples. (**E**) The expression of miR-634 in glioma cells transfected with si-DANCR or si-NC. (**F**) The expression levels of DANCR and miR-634 were enriched in Ago2 immunoprecipitates relative to control IgG immunoprecipitates; *P<0.05.

Accumulating evidence suggested that lncRNA might function as ceRNAs to regulate miRNAs and hence functionally liberates other RNA transcripts. To examine whether DANCR has a similar mechanism in glioma, we used bioinformatics software DIANA to predict the potential miRNA binding sites in DANCR. Results demonstrated that miR-634 formed complementary base pairing with DANCR (Figure 3A). Dual-luciferase assay showed that miR-634 mimics decrease the luciferase activity of DANCR-Wt, while it may not affect the luciferase activity of DANCR-Mut (Figure 3B; *P*<0.05). To further test this prediction, we determined the expression of miR-634 in 47 glioma tissue samples. Results showed that miR-634 was significantly decreased in glioma tissues and inversely correlated with DANCR expression in glioma tissues (Figure 3C and D; *P*<0.05). Moreover, we explored miR-634 expression in DANCR knockdown glioma cells. Results showed that miR-634 expression was significantly increased in si-DANCR transfected glioma cells (Figure 3E; *P*<0.05). In addition, an RNA-binding protein immunoprecipitation (RIP) assay was performed to determine whether DANCR and miR-643 were in the same RNA-induced silencing complex (RISC). Results showed that DANCR and miR-643 were higher in anti-Ago2 group than that in anti-normal IgG group (Figure 3F; *P*<0.05).

### miR-634 inhibitors reversed the effect of DANCR knockdown in glioma cells

**Figure 4:**
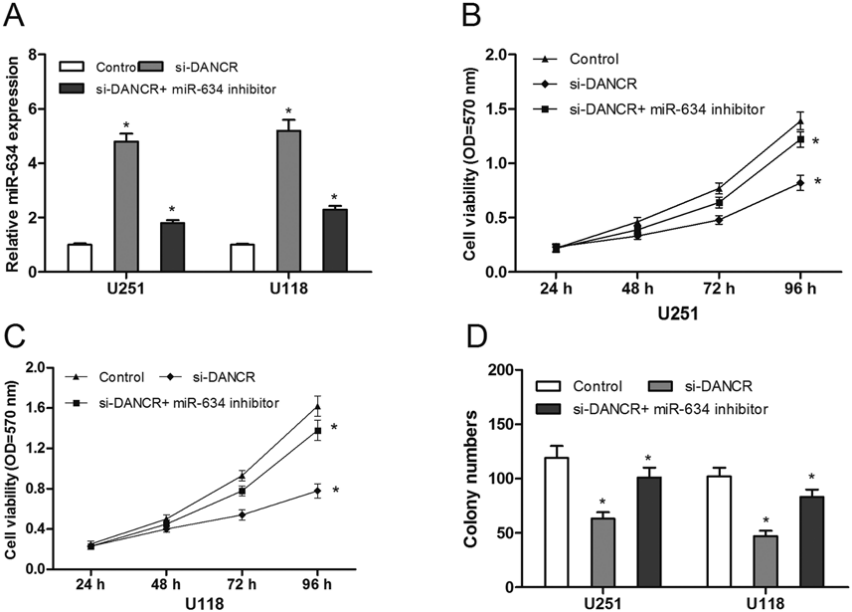
miR-634 inhibitors reversed the effect of DANCR knockdown on glioma cells (**A**) The expression of miR-634 in glioma cells transfected with miR-634 inhibitor or si-DANCR was determined by qRT-PCR. (**B and C**) The cell proliferation of glioma cells transfected with miR-634 inhibitor or si-DANCR was determined by CKK-8 assay. (**D**) Colony numbers of glioma cells transfected with miR-634 inhibitor or si-DANCR were determined by colony formation assay; *P<0.05.

To explore whether DANCR exerted biological functions through miR-634, we reduced expression miR-634 in si-DANCR transfected glioma cells (Figure 4A; *P*<0.05). CCK-8 assay showed that miR-634 inhibitors attenuated the suppressing proliferation effect of DANCR knockdown in glioma cells (Figure 4B and C; *P*<0.05), which was also observed in colony formation assay (Figure 4D; *P*<0.05). These results indicated that DANCR inhibition suppressed glioma cells growth partly via regulating miR-634 expression.

### DANCR modulated RAB1A expression via miR-634

**Figure 5:**
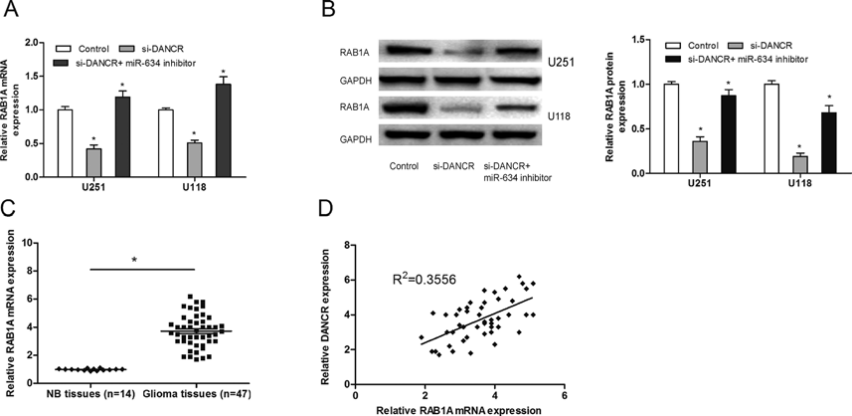
DANCR modulated RAB1A expression via miR-634 (**A**) The mRNA expression of RAB1A in glioma cells after transfected with si-DANCR or miR-634 inhibitor. (**B**) The protein expression of RAB1A in glioma cells after transfected with si-DANCR or miR-634 inhibitor. (**C**) The expression of RAB1A in glioma tissue samples and NB tissue samples was determined by qRT-PCR. (**D**) Positive correlation between DANCR expression and RAB1A expression in 47 glioma tissue samples; *P<0.05.

It has been found that miR-634 functions as a tumor suppressor in human hepatocellular carcinoma by down-regulating RAB1A [13]. To determine whether DANCR could function as a ceRNA for RAB1A via modulating miR-634 in glioma, we determined the mRNA and protein levels of RAB1A after glioma cells transfected with si-DANCR combined with miR-634 inhibitor. Results showed that DANCR inhibition suppressed RAB1A expression in glioma cells both at mRNA and protein levels, and miR-634 inhibitor restored the reduction of RAB1A expression in DANCR suppressed glioma cells (Figure 5A and B; *P*<0.05). Additionally, we determined the expression of RAB1A in glioma tissue samples by qRT-PCR, results showed that RAB1A expression was significantly increased and positively associated with DANCR expression in glioma tissues (Figure 5C and D; *P*<0.05). Taken together, these results indicated that DANCR could act as a sponge for miR-634 to up-regulate the expression of RAB1A in glioma.

## Discussion

Glioma is the most common and aggressive malignant tumor of the central nervous system and has a high rate of recurrence and mortality [15]. Although there have been advances in multimodal treatments such as surgery, radiotherapy and chemotherapy, overall survival of most patients with glioma remains dismal, especially in cases of glioblastoma [2,16]. Recently, lots of studies showed that lncRNAs play considerable functional roles in glioma progression [17,18].

Significantly different lncRNA profiles could serve as phenotypic signatures for different cancers for their exploitation in cancer prognostics and therapeutics [19]. Recently, many studies demonstrated that DANCR might act as an oncogenic lncRNA in tumor progression. For example, Liu et al. [20] showed that overexpression of lncRNA DANCR was associated with advanced tumor progression and poor prognosis in patients with colorectal cancer. Jia et al. [21] found that lncRNA DANCR promoted invasion of prostate cancer through epigenetically silencing expression of TIMP2/3. Jiang et al. [22] indicated that lncRNA DANCR promoted tumor progression and cancer stemness features in osteosarcoma by up-regulating AXL via miR-33a-5p inhibition. In the present study, we found that DANCR expression was markedly increased and positively associated with advanced tumor grade in glioma patients. Down-regulated expression of DANCR inhibited glioma cells growth and arrested cell cycle in G0/G1 phase. Our results indicated that DANCR could act as an oncogenic lncRNA in glioma progression.

Increasing studies showed that lncRNA could act as competing endogenous RNA (ceRNA). These RNA transcripts sponge and suppress miRNAs, and alleviate their inhibitory effect on target genes of the miRNAs [23,24]. For example, Lu et al. [25] showed that lncRNA BC032469 act as a novel ceRNA up-regulated hTERT expression by sponging miR-1207-5p and promoted proliferation in gastric cancer. Huang et al. [26] found that lncRNA CASC2 functioned as a ceRNA by sponging miR-18a in colorectal cancer. Fang et al. [27] suggested that lncRNA HNF1A-AS1 mediated repression of miR-34a/SIRT1/p53 feedback loop promoted the metastatic progression of colon cancer by functioning as a ceRNA. However, whether DANCR affected glioma progression by regulating miRNAs has not yet been reported.

In the present study, our data showed that miR-634 acted as an inhibitory target of DANCR by bioinformatic analysis and luciferase reporter assay. QRT-PCR showed that the expression of miR-634 was decreased in glioma tissue samples and inversely corrected with DANCR expression. In addition, our data showed that miR-634 expression was significantly increased in si-DANCR transfected glioma cells. RIP assay showed that DANCR and miR-643 were higher in anti-Ago2 group than that in anti-normal IgG group. Furthermore, we found that miR-634 inhibitor could reverse the effect of DANCR knockdown on glioma cells. Taken together, these findings suggested that miR-634 in glioma cells was regulated by lncRNA DANCR.

RAB1A is a small GTPase well known for its role in regulating ER-to-Golgi vesicular transport [28]. It is a highly conserved protein, which has been identified in 158 different organisms, ranging from yeast to humans [29]. Recently, lots of studies indicated that RAB1A plays important roles in tumor progression. For example, Wang et al. [30] showed that expression of RAB1A was up-regulated in human lung cancer and associated with tumor size and T stage. Xu et al. [31] showed that inhibition of RAB1A suppressed epithelial–mesenchymal transition and proliferation of triple-negative breast cancer cells. Yang et al. [32] found that miR-15b-5p induced endoplasmic reticulum stress and apoptosis in human hepatocellular carcinoma, both *in vitro* and *in vivo*, by suppressing RAB1A. However, the interaction between lncRNA and RAB1A in glioma remains unclear. In the present study, our data showed that DANCR inhibition suppressed RAB1A expression in glioma cells, and miR-634 inhibitors restored the reduction of RAB1A expression in DANCR suppressed glioma cells. Furthermore, we found RAB1A was up-regulated in glioma tissue samples and positively associated with the expression of DANCR.

In summary, our studies indicated that DANCR promoted glioma progression by functioning as miR-634 sponge, and indicated a novel DANCR-miR-634-RAB1A signaling pathway regulatory network in glioma. These findings suggested that DANCR could be a potential therapeutic target in glioma treatment.

## Abbreviations

ceRNA: competing endogenous RNA
DANCR: differentiation antagonizing nonprotein coding RNA
lncRNA: long noncoding RNA
NHA: normal human astrocytes
PI: propidium iodide
RIP: RNA immunoprecipitation
RISC: RNA-induced silencing complex
